# Plasmonic Enhancement of Selective Photonic Virus Inactivation

**DOI:** 10.1038/s41598-017-12377-5

**Published:** 2017-09-20

**Authors:** Mina Nazari, Min Xi, Sarah Lerch, M. H. Alizadeh, Chelsea Ettinger, Hisashi Akiyama, Christopher Gillespie, Suryaram Gummuluru, Shyamsunder Erramilli, Björn M. Reinhard

**Affiliations:** 1 Departments of Electrical and Computer Engineering, Boston University, Boston, MA 02215 United States; 2 Departments of Physics, Boston University, Boston, MA 02215 United States; 3 Departments of Chemistry, Boston University, Boston, MA 02215 United States; 4 The Photonics Center, Boston University, Boston, MA 02215 United States; 5 Department of Microbiology, Boston University School of Medicine, Boston, MA 02118 United States; 6 Next Generation Bioprocessing, Millipore-Sigma, Bedford, MA 01730 United States

## Abstract

Femtosecond (fs) pulsed laser irradiation techniques have attracted interest as a photonic approach for the selective inactivation of virus contaminations in biological samples. Conventional pulsed laser approaches require, however, relatively long irradiation times to achieve a significant inactivation of virus. In this study, we investigate the enhancement of the photonic inactivation of Murine Leukemia Virus (MLV) *via* 805 nm femtosecond pulses through gold nanorods whose localized surface plasmon resonance overlaps with the excitation laser. We report a plasmonically enhanced virus inactivation, with greater than 3.7-log reduction measured by virus infectivity assays. Reliable virus inactivation was obtained for 10 s laser exposure with incident laser powers ≥0.3 W. Importantly, the fs-pulse induced inactivation was selective to the virus and did not induce any measurable damage to co-incubated antibodies. The loss in viral infection was associated with reduced viral fusion, linking the loss in infectivity with a perturbation of the viral envelope. Based on the observations that physical contact between nanorods and virus particles was not required for viral inactivation and that reactive oxygen species (ROS) did not participate in the detected viral inactivation, a model of virus inactivation based on plasmon enhanced shockwave generation is proposed.

## Introduction

Pulsed lasers provide new opportunities for imaging or modulating cellular behavior in a diverse range of diagnostic^1,2^ and therapeutic^3^ applications. In particular, virus inactivation through exposure to ultrashort laser pulses is emerging as a potential alternative to existing biocides and ionizing radiation techniques^4–7^. The current interest in photonic virus inactivation derives from the need for new technologies that achieve a selective inactivation of the pathogens in the presence of other biomolecules or even living cells in food, feed stock in pharmaceutical bioreactors, therapeutic compounds and other sensitive areas with relevance for human and animal health. For many of these applications, harsh chemical or ionizing radiation techniques are not appropriate as they lack sufficient selectivity. Importantly, Kong-Hon Tsen and coworkers have demonstrated in a series of elegant studies that irradiation with femtosecond (fs) laser pulses with visible (425 nm)^4–8^ or near-Infrared (776 nm^9–11^, 850 nm^12^) wavelengths results in an inactivation of both enveloped and non-enveloped viruses under conditions that do not impact mammalian cells^13^. Tsen and coworkers postulated that fs laser induced virus inactivation is a non-thermal effect that results from impulsive stimulated Raman scattering (ISRS) driven breaking of non-covalent bonds in the virus^6,10^. Although individual broken contacts can reform, the authors argued that an excessive bond-breaking results in an irreversible loss of structural integrity of virus particles^4,6^ and potentially in viral protein aggregation^5^. Theoretical analysis revealed that for ISRS to be effective, high laser intensities are required^14^. Under these conditions alternative mechanisms to drive structural and chemical transformations exist, including multiphoton absorption. It also deserves mentioning that a different group was unable to reproduce the inactivation of phages through fs laser irradiation^15^, highlighting the need for further research into the mechanism of inactivation.

To date, photonic inactivation was successfully achieved with focused fs laser beams and relatively long irradiation times of ≥1 h^10^. The need for long irradiation times limits the scalability of the photonic virus inactivation process and impedes the practical implementation of photonic virus inactivation strategies in many cases.

In this manuscript, we explore the plasmonic enhancement of photonic virus inactivation as a possible strategy to overcome some of the challenges associated with pulsed laser driven virus inactivation. Since noble metal nanoparticles convert incident electromagnetic waves into localized charge density oscillations, so called localized surface plasmon resonances (LSPRs)^16^, they generate high local E-field enhancements in electromagnetic hot-spots^16,17^. The strong E-field generated by the plasmonic nanoparticles can enhance the previously discussed virus inactivation mechanisms and, as we show in this manuscript, facilitate new virus inactivation processes. We demonstrate that resonant nanoparticles whose LSPR overlap with the excitation pulse, enhance the virus inactivation but that non-resonant nanoparticles have no effect. The effect of resonant plasmonic nanoparticles on virus inactivation as function of laser power when irradiated for a short exposure time of 10 s is characterized in detail. Since both efficiency and selectivity are important figures of merit for photonic virus inactivation, we monitored the selectivity towards virus particles by measuring the functionality of IgG antibodies co-incubated with the virus particles during laser exposure. Our data indicate that the plasmonically enhanced photonic inactivation is highly selective towards virus particles and generates no detectable collateral damage to the antibodies, and that the mechanism of inactivation differs from those of previously established photocatalytic^18^ and photothermal^19^ inactivation methods.

## Methods and Materials

### Laser Set-Up

The ultrashort pulsed (USP) excitation source used in this study is a femtosecond laser based upon a Legend Elite Duo (Coherent Inc.) Ti-sapphire regenerative amplifier. The laser produces a continuous train of 35 fs pulses at a repetition rate of 1 kHz centered at 805 nm with energies up to 7.5 mJ and spectral width of about 25 nm. The laser beam was incident on a quartz cuvette containing 250 µL of virus sample. Different laser powers on the sample were obtained using beam splitters and directing only a portion of the total laser beam to the sample area. The USP laser spot size was approximately 1 cm^2^ and the typical exposure time of the sample to the laser irradiation was 10 sec. All laser irradiation studies were carried out at 22 °C. After irradiation, the samples were immediately stored at 4 °C.

### Photoacoustic Measurements

**Figure 1:**
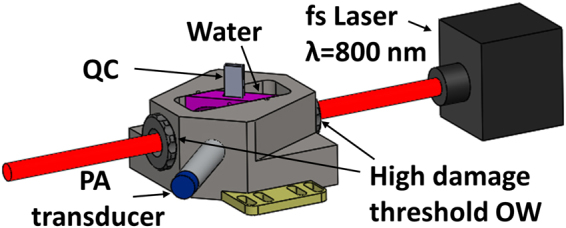
Schematic of experimental setup for photoacoustic measurements. The following abbreviation are used: fs: femtosecond, QC: quartz cuvette, PA: photoacoustic, and OW: optical window.

The acoustic signal generated by laser irradiated nanorods was collected in a homebuilt ultrasound detector. Figure 1 depicts the acoustic wave measurement setup, based on the design of Yelleswarapu and co-workers^20–22^. The nanorod sample was placed in a 1 mm quartz cuvette, which was located in a custom cell filled with water for ultrasound coupling. The cuvette was oriented at an angle of 45° relative to the 0.3 W fs laser beam. The photoacoustic signal (ultrasonic wave) was collected using a 2.25 MHz focused water immersion transducer.

### Nanorod Synthesis and Functionalization

We used the seed-mediated growth technique described by Vigderman *et al*. to synthesize gold nanorods^23^. 460 μL of a freshly prepared solution of 0.01 M sodium borohydride dissolved in 0.01 M sodium hydroxide was rapidly injected into 10 mL of 0.5 mM HAuCl_4_ solution containing 0.1 M CTAB under extensive stirring. A change of color from greenish to light brown indicated the successful formation of gold nanoparticle seeds. To synthesize gold nanorods with a longitudinal resonance at ~800 nm, 11.5 μL of 0.1 M silver nitrate solution was added to 10 mL of 0.5 mM HAuCl_4_ solution containing 0.1 M CTAB. Subsequently, 500 μL of 0.1 M aqueous hydroquinone solution was added, and the resulting mixture was hand-stirred until it became clear. Then, 160 μL of gold seed solution was added under thorough stirring. The resulting mix was allowed to age overnight. To obtain PEGylated nanorods, 2.5 µL of 10 mM PEG2 (HS—CH_2_CH_2_—(C_2_H_4_O)_77_—N_3_) were added together with 2.5 µL of 10 mM HS-(CH_2_)_11_-(CH_2_CH_2_O)_6_OCH_2_-COOH to 1 mL of gold nanorods in the presence of 3% v/v of Tween^®^ 20. The samples were incubated overnight and washed by centrifugation and subsequent resuspension. Alkyne-functionalized annexinV was cross-linked to the azide functionalized nanorods in the presence of 500 μM ascorbic acid and 100 μM CuSO_4_ *via* Cu^I^ catalyzed click-reaction^24^. The nanorods were cleaned again through repeated centrifugation and resuspension in DDI water or buffer.

### Cells and Virus Production

HEK293T and Rat-2 cells were maintained in DMEM supplemented with heat-inactivated 10% fetal bovine serum (Invitrogen) and Pen/Strep (HyClone). Luciferase expressing, single cycle of replication competent MLV particles (MLV/luc) were derived via co-transfection of HEK293T cells with pLNC-luc (luciferase expressing retroviral expression vector) and pCL-Eco (MLV packaging vector that expresses MLV Gag, Pol and ecotropic Env glycoproteins; Imgenex) plasmids, as previously described^25^. For the virus fusion assay described below, HEK293T cells were co-transfected with pLNC-luc, pCL-Eco and S15-BlaM plasmids. To generate the S15-BlaM expression plasmid that expresses a Src-eptiope tagged β-lactamase fusion protein upon transfection, the N-terminal 15 amino acid sequence of c-Src (S15) was cloned in frame upstream of β-lactamase orf in pcDNA3.1/zeo + (Invitrogen) eukaryotic expression plasmid. S15-BlaM is incorporated into MLV particles upon virus particle budding from the plasma membrane of virus-producing cells^26,27^. Virus-containing supernatants were harvested 2 days post transfection, and passed through 0.45 µm filters. Virus was concentrated by ultracentrifugation on a 20% sucrose cushion [28,000 rpm at 4 °C for 2 hours with a SW32 Ti rotor (Beckman Coulter)]. Virus pellets were resuspended in PBS, aliquoted, and stored at −80 °C until further use.

### Preparation of Nanorod-Virus Samples

PEGylated nanorods or nanorods loaded with annexinV were centrifuged at 5000 rpm for 10 min. The supernatant was discarded and 17 µL of the pellet was added to 50 µL of MLV/luc, followed by overnight incubation at 4 °C. Then, 500 µL of IgG antibody (anti-p24^gag^ monoclonal antibody; Clone 183-H12-5C; NIH AIDS Research and Reference Reagent Program, contributed by Dr. Bruce Chesebro) in RPMI medium was added to the mixture and the mixture was diluted by adding 1x Tris (pH 7) or PBS (pH 7.4) buffer to final volume of 1 ml. The nanorod to virus ratio was more than 10000:1. The concentration of the nanorods was quantified using a UV-vis spectrometer (Cary 5000 spectrophotometer).

### ROS Scavengers

A solution containing three different ROS scavengers were used. Sodium azide (NaN_3_) and Manitol were dissolved in 20 mM Tris (pH 7.0). Then Manganese (III) Tetrakis (4-benzoic acid) Porphyrin (MnTBAP) dissolved in 0.1 M NaOH was added. This solution was subsequently added to virus samples containing antibodies from a hybridoma preparation or to mixtures containing virus, PEGylated nanorods, and antibodies. All samples were diluted with Tris buffer. The final scavenger concentrations were 10 mM Manitol, 10 mM Sodium azide and 0.02 mM MnTBAP. The pH of the final solution was 7.

### ELISA

To test the efficacy of the fs laser inactivation protocol on functionality (epitope recognition) of a monoclonal antibody, an anti-p24^gag^ monoclonal antibody was exposed in the presence or absence of MLV/luc particles to fs laser, as described above. A sandwich ELISA was performed to test the ability of the anti-p24^gag^ monoclonal antibody to quantify standard amounts of HIV-1 p24^gag^ antigen, as described previously^28^. Briefly, 2-fold dilutions of p24^gag^ antigen (ABI, Inc) was bound to HIV-Ig (NIH AIDS Research and Reference Reagent Program, contributed by NABI and National Heart Lung and Blood Institute, Dr. Luiz Barbosa) coated wells and detected with unexposed control or laser exposed anti-p24^gag^ monoclonal antibody, and HRP-conjugated goat anti-mouse secondary antibody (Sigma). ELISA was developed with a peroxidase substrate (KPL, Inc) and the standard curves were generated with each of the control (untreated) or fs laser-exposed anti-p24^gag^ monoclonal antibodies.

### Virus Fusion Assay

To investigate MLV/luc fusion to Rat2 target cells, a FACS based assay^29^ was utilized with some modification. Briefly, untreated or laser-exposed MLV/luc particles containing S15-BlaM fusion protein was used to infect target cells. After incubating 2 hours at 37 °C, cells were washed with CO_2_-independent media (Invitrogen) and incubated in CCF2 (fluorogenic substrate of β-lactamase)-containing media at 18 °C over night, washed, fixed with 4% PFA. The number of β-lactamase positive cells was determined by FACS analysis using a LSRII flow cytometer (BD).

### Quantification of Virus Inactivation

Virus inactivation was determined by infecting cells (mouse fibroblast cell line) with treated (laser-inactivated) or untreated viruses. Cells were then incubated for 48 h *prior* to lysis. The lysates were then used for measurement of luciferase activity using a chemiluminescent reporter (Bright-Glo, Promega). The ratio of the intensity of the luminescence to a calibration standard is measured in RLU (relative light units), and is a linear function of the reporter concentration. If RLU_*c*_ and RLU_*s*_ are the measured RLU values of the control and sample respectively, the Log-Reduction Value (LRV) defined as LRV = log_10_(RLU_*c*_/RLU_*s*_) gives a quantitative measure of the extent of virus infection in the culture.

### Quantification of Cell viability

In order to test for potential cytotoxicity of nanorods, laser irradiated and non-irradiated nanorods media were harvested and incubated with Rat-2 cells at 37 °C overnight. The Trypan blue exclusion test was then used to determine the percentage of viable cells.

### Gel Electrophoresis of Nanorods

Nanorod samples were run on 2% agarose gels made from 0.5x TBE. The same buffer was used as running buffer. The samples were loaded with ficol and were run with a current of 200 mA, voltage of 140 V for 30 min.

### Statistical Analysis

The variability in the data were analyzed by Anova with subsequent post-hoc test as implemented in Matlab. Differences between samples with a significance level of P < 0.05 are marked with one asterisk (*), and for P < 0.01 with two asterisks (**). NS was used to mark non-significant differences.

### Data availability Statement

The datasets generated during and/or analyzed in the current study are available from the corresponding authors upon request.

## Results and Discussion

We chose Murine Leukemia Virus (MLV) as target for our viral inactivation studies due to its similarity with endogenous mouse retroviruses that are known contaminants in the biopharmaceutical industry^30,31^. All experiments were performed with a recombinant virus that expresses the luciferase reporter gene upon establishment of productive infection in target cells, providing a robust strategy for the quantification of virus infectivity. In a first set of experiments we tested the hypothesis that nanorods whose LSPR overlaps with the incident fs laser pulse enhances the efficacy of light-induced laser activation. To that end, we compared the viral infectivity obtained with virus particles exposed to fs laser irradiation with constant pulse duration but different average powers. Importantly, we kept the exposure time of the individual samples constant at 10 s, which is 3 orders of magnitude shorter than the exposure time in all previous photonic virus inactivation studies.

### Nanorods for Viral Inactivation

**Figure 2:**
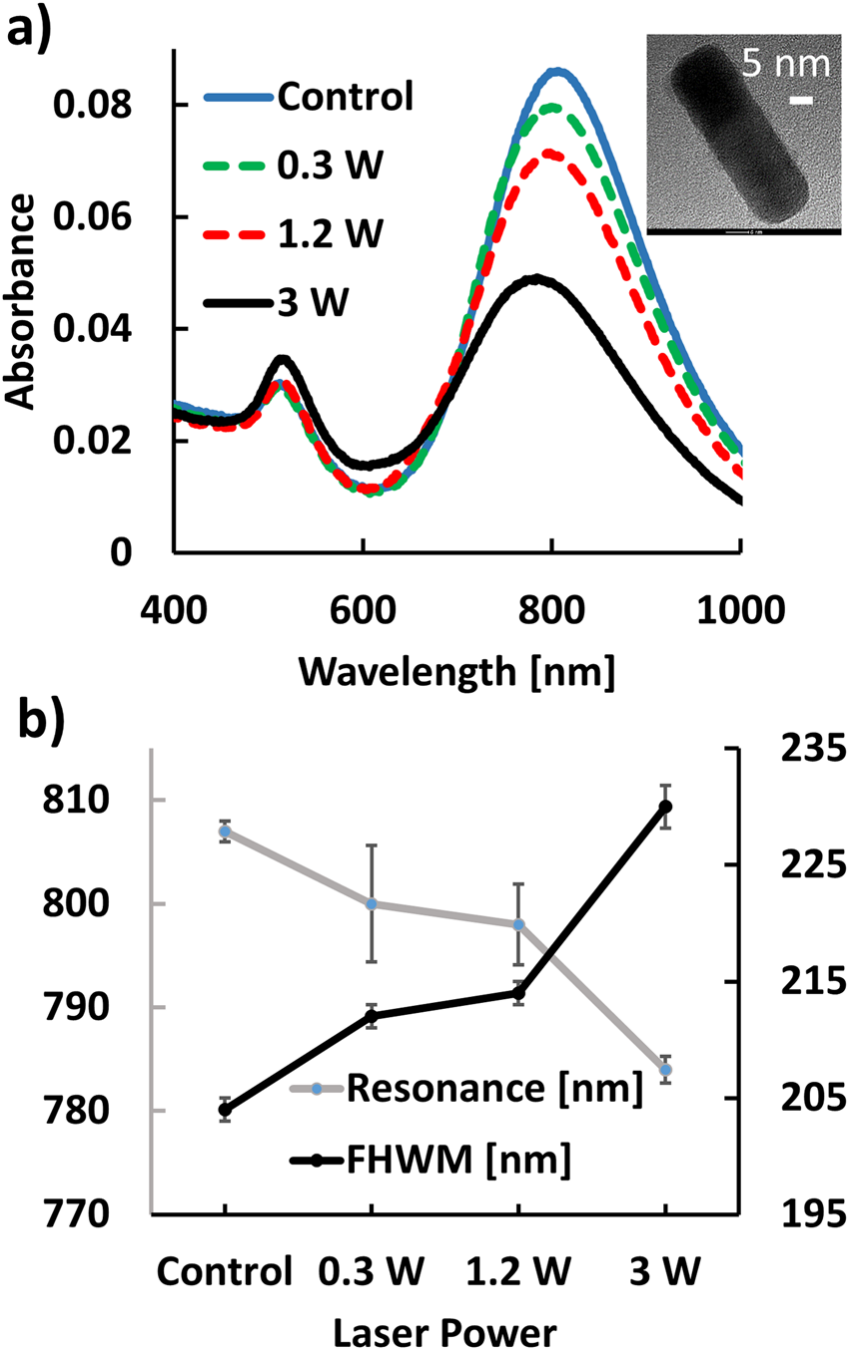
(**a**) UV-Vis spectra of nanorods recorded after 10 s of fs laser irradiation with different average powers. Control refers to rods without irradiation. Inset: TEM image of nanorod. Size bar = 5 nm. (**b**) Peak wavelengths (gray) and widths (black) of the longitudinal nanorod plasmon mode as function of the average incident laser power.

The LSPRs of gold nanoparticles are strongly morphology dependent, and the aspect ratio of nanorods represents a rational control parameter to tune the plasmon resonance across the visible range of the electromagnetic spectrum to the Near-Infrared (NIR) and beyond^32,33^. We used gold nanorods with an aspect ratio of around 4, with typical lengths of ~60 nm. We found that these nanorods sustain a longitudinal plasmon mode that peaks close to the wavelength of the fs laser emission (805 nm) as is shown in the UV-Vis spectra of the nanorod “control” (no laser exposure) in Fig. 2a. A TEM image of a representative nanorod is provided as inset. The gold nanorods were obtained through a surfactant-directed synthesis^34^ using cetrimethylammonium bromide (CTAB) as ligand, which favors growth along the crystallographic [110] axis. Due to the functionalization of the nanorod surface with CTAB, the nanorods have a positive zeta-potential of approximately ζ =  + 23 mV. As CTAB is cytotoxic, we replaced it with polyethyleneglycol (PEG) ligands. In a first set of experiments we tested the stability of the nanorods when irradiated with 805 nm laser pulses with 35 fs duration for 10 s. We varied the average laser power between 0.3 W, 1.2 W and 3 W and recorded UV-Vis spectra after laser irradiation. These spectra and the spectrum of the non-irradiated control sample are summarized in Fig. 2a. The UV-Vis spectra contain two characteristic peaks. One is centered at 520 nm and is assigned to the transverse plasmon mode of the nanorods, whereas the second peak at 800 nm is assigned to the longitudinal plasmon mode^32^. The peak wavelength of the longitudinal mode intensities and the associated full width at half maximum (FWHM), are plotted in Fig. 2b. Interestingly, we observe a blue shift and broadening of the longitudinal mode. These effects are accompanied by a decrease in the spectral intensity of the longitudinal mode and an increase in the intensity of the transverse mode (Fig. 2a).

**Figure 3:**
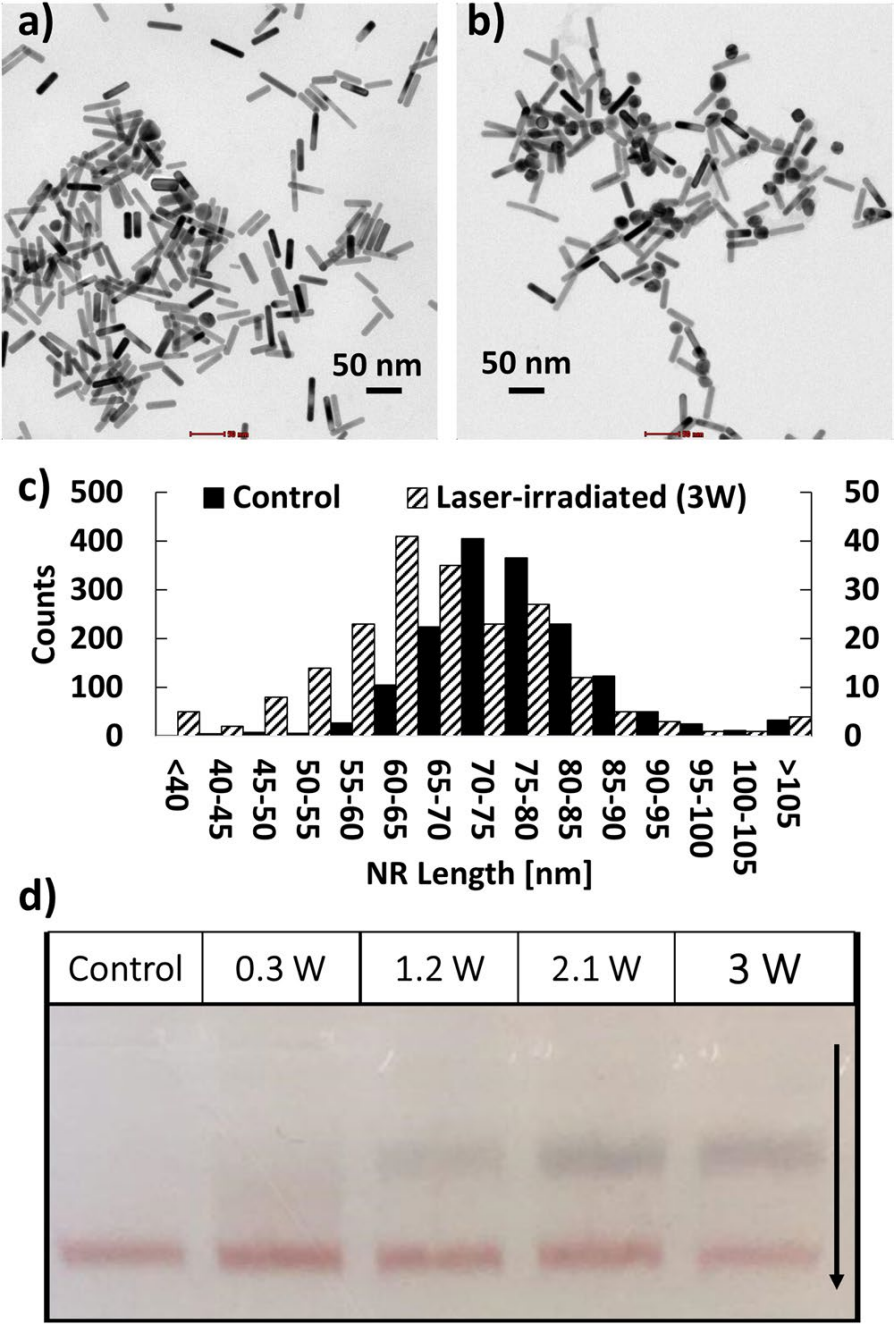
TEM image of nanorods before (**a**) and after (**b**) irradiation with 35 fs laser pulses for 10 s (average power 3 W). (**c**) Histogram of nanoparticle length before (control) and after laser irradiation. (**d**) 2% Agarose gels of rod samples collected before (control) and after laser irradiation with specified average powers. The formation of a second band at high laser powers indicates agglomeration.

The observed spectral changes can be attributed to two main effects. Surface melting^35,36^ of the nanorods leads to shorter and wider rods with increased oscillator strength for the transverse plasmon mode and a blue-shifted longitudinal plasmon resonance. Early studies experimentally showed that an average of ~60 femtojoule (fJ) energy, which is close to our experimental condition, is required to melt a single gold nanorod with an aspect ratio ~4^37^. Fig. 3a and b shows representative TEM images of nanorods before and after laser irradiation with an average power of 3 W, respectively. Visual inspection of the two images suggests that after laser irradiation the sample contains significantly more spheroidal particles. The histogram of the lengths of the nanorods in Fig. 3c confirms a systematic shortening of the nanorods in response to laser irradiation. The second effect contributing to the spectral shift is an agglomeration of the nanorods due to fs laser irradiation. Figure 3d shows a 2% agarose gels of nanorods irradiated with different laser powers. At low power, the gel contains only the nanorod band, but for powers ≥1.2 W a second slower band of dark grey color is detected at the cost of intensity for the original nanorod band. This new slower moving band contains agglomerates of nanorods. This redistribution of relative intensity from the nanorod band into the agglomeration band continues if the average laser power is further increased to 3 W. Agglomeration of nanorods leads to the formation of new hybridized plasmon modes that – depending on the geometry of the clusters – can be both blue- or red-shifted with regard to the longitudinal mode of the individual nanorod. A side-by-side arrangement, for instance, leads to a systematic blue-shift in the optically active plasmon mode, whereas end-to-end, T-shape, and off-scale side-by-side arrangements yield red-shifts^38^. The different spectral responses account for the broadening of the plasmon band centered at around 800 nm in the nanorod spectra. The observed agglomeration indicates a decomposition of the nanorod surface ligands under pulsed laser irradiation that results in a decrease of colloidal stability.

Although the nanorods show some morphological restructuring and agglomeration, we emphasize that both the UV-Vis data and the gel studies confirm that the sample still contain a substantial fraction of resonant nanorods under the chosen irradiation conditions.

### Plasmonically Enhanced Virus Inactivation

In a typical virus inactivation experiment, PEGylated nanorods were incubated with MLV particles in a ratio of more than 10000:1 in a total volume of 67 μL at 4 °C for up to 12 h. The samples were then diluted by adding 933 μL of PBS, pH 7.4 to a total volume of 1 mL. The final virus concentration was such that infections of Rat2 reporter cells with the luciferase encoding recombinant viruses resulted in approximately 1 × 10^6^ relative light units (RLU). We performed the inactivation studies with PEGylated nanorods as well as with rods functionalized with annexinV that binds to phosphatidylserine (PS) of the enveloped MLV. For the latter, Ca^2+^ was added to the binding buffer when incubating annexinV-functionalized nanorods with virus particles to achieve a final Ca^2+^ concentration of 2 mM.

In many applications it is necessary to inactivate the virus in a complex biological matrix. For instance, virus inactivation plays a critical role in the pharmaceutical industry for the fabrication of monoclonal antibodies (mAbs). We emulated realistic process conditions by performing the virus inactivation in an IgG antibody (total protein concentration: 160 µM) containing Tris solution as biological matrix. The proteins were added after overnight co-incubation of nanorod and virus.

**Figure 4:**
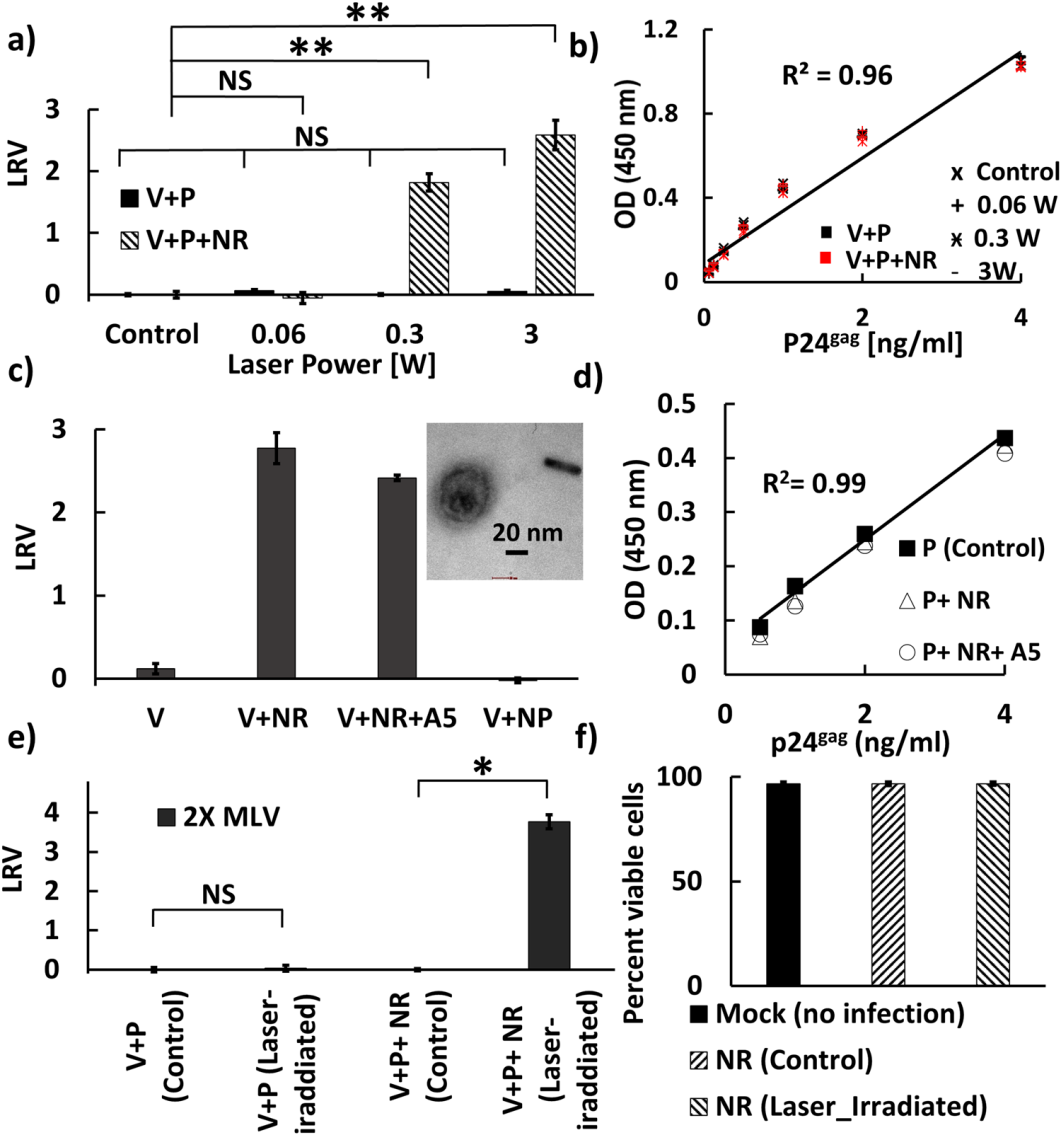
(**a**) Virus log-reduction-value (LRV) LRV measured for virus (V) and virus + PEGylated nanorods (NR) both in the presence of IgG antibody (P) as function of average 805 nm laser power. Laser pulse duration was 35 fs. The irradiation time was held constant at *t* = 10 s. Control = no laser irradiation. (**b**) Binding curves for the IgG antibody (P) recovered from the experiments in (**a**) in the presence (red) and absence (black) of nanorods as determined by ELISA. (**c**) LRV obtained for virus (V) alone, virus + PEGylated nanorods (NR), virus + annexinV functionalized nanorod (NR+A5) and virus + spherical nanoparticles (NP) after irradiation to a pulsed (35 fs) 805 nm laser with an average power of 3 W for 10 s exposure time. Inset: TEM image of laser irradiated virus + PEGylated nanorod. Size bar = 20 nm. (**d**) Binding curves for antibody (P) irradiated with the pulsed 805 nm laser for 10 s at an average power of 3 W as determined by ELISA. Control = no laser irradiation (**e**) LRV obtained under similar laser conditions as in (**c**) but with two-fold increased virus concentration. The increase in LRV with virus concentration indicates that the LRV values are limited by the sensitivity of the infection assay (**f**) Percentage of viable cells for no-treatment control (mock), cells after incubation with a nanorod solution that was not exposed to the laser, and cells treated with a nanorod solution that was exposed to the laser. Laser irradiation conditions were identical to (**e**).

In a first set of experiments, we tested the hypothesis that resonant plasmonic nanorods enhance fs pulsed laser driven virus inactivation. To that end, virus samples were irradiated for 10 s with 805 nm laser pulses with a width of 35 fs in the presence and absence of pegylated nanorods. All samples contained IgG antibodies. Figure 4a shows the measured LRV values for average powers of 0 W (control), 0.06 W, 0.3 W, and 3 W. While in the absence of PEGylated nanorods none of these average powers resulted in a measurable LRV, after addition of PEGylated nanorods average powers ≥0.3 W were sufficient to generate a highly significant LRV. A LRV = 1.8 was obtained for an average power of 0.3 W and the viral inactivation further enhanced to LRV = 2.6 for an average power of 3 W. The striking difference in virus inactivation between samples with and without PEGylated nanorods provides first experimental evidence of a strong enhancement of photonic inactivation through PEGylated nanorods. To assess the potential collateral damage that laser irradiation has on the co-incubated IgG antibodies, we compared the binding of the recovered IgG antibodies to its epitope using ELISA (Fig. 4b). Intriguingly, our ELISA studies revealed essentially identical binding curves for antibodies exposed to 0.06 W, 0.3 W, or 3 W of laser irradiation and for the no irradiation control. The absence of any laser-induced changes in binding affinity is remarkable and argues against any systematic collateral damage during virus inactivation.

In a second set of experiments, we used the maximum average laser power of 3 W and measured viral inactivation in the presence of three different nanoparticles: virus + PEGylated nanorods; virus + annexinV-functionalized nanorods; virus + spherical nanoparticles. Unlike for the nanorods, the LSPR of the 40 nm diameter gold nanoparticles (530 nm) did not overlap with the exciting laser pulse. The nanorod and nanoparticle to virus ratios were both higher than 10000:1. Additional controls included laser irradiation of viruses without nanoparticles. After laser irradiation for 10 s, virus infectivity was quantified (Fig. 4c). The 805 nm fs laser irradiation had no effect on virus infectivity in the absence of nanorods, or upon incubation of virus with spherical nanoparticles whose resonance does not overlap with the incident laser pulse. In contrast, addition of PEGylated nanorods whose longitudinal mode lies close to 800 nm led again to a strong reduction in viral infectivity (LRV~2.7). Interestingly, the LRV value obtained with PEGylated nanorods functionalized with annexinV, which can bind to phosphatidylserine (PS) in the viral membrane, was not higher than that for the PEGylated nanorods. Since the PEGylated nanorods used in our experiments did not show any systematic cytotoxicity before or after laser radiation (Fig. 4f), we conclude that the decrease in infectivity observed for virus samples irradiated with the pulsed laser in the presence of resonant gold nanorods results from an enhancement of photonic virus inactivation through resonant plasmonic nanorods.

We tested for potential collateral damage to IgG antibodies through laser irradiation (3 W, 10 s) of PEGylated and annexinV functionalized nanorods through ELISA (Fig. 4d). This time, the experiments were performed in the absence of the virus. Again, we observed essentially identical binding curves for the antibody before and after laser irradiation, in the absence or presence of the PEGylated nanorods, respectively (Fig. 4d). The antibody binding affinity was not significantly affected by the NIR fs laser irradiation of resonant nanoparticles under conditions that achieved a very significant inactivation of virus particles.

One unexpected finding from Fig. 4c is that annexinV functionalization of the nanorods does not yield a stronger reduction of infectivity when compared with the PEGylated nanorods. In fact, the PEGylated nanorods without any virus binding functionality achieved slightly higher LRV values. To check for non-specific binding of PEGylated nanorods to MLV particles we imaged the mix of virus and PEGylated nanorods in the TEM (Figure S1). We did not detect any significant spatial colocalization between virus particles and PEGylated nanorods, indicating that the non-specific binding of the PEGylated nanorods to the virus particles is low. A magnified TEM image of a virus particle is included as inset in Fig. 4c.

The fact that annexinV fails to enhance virus inactivation and the absence of a significant non-specific binding to MLV in the case of the PEGylated nanorods suggests that a direct binding between nanorods and virus particles is not required for the nanorod-enhanced photonic virus inactivation.

The LRVs measured in Fig. 4a and c are limited by a relatively low virus titer, which was close to the detection limit of the luciferase activity assay used for the quantification of virus infection. Higher LRVs can be achieved by increasing the amount of initial virus input in the system. Indeed, when we increased the virus concentration by a factor of 2 and used twice as much sample volume for virus infection as before, we measured a LRV = 3.76 (Fig. 4e). As our analysis revealed that no binding between nanorod and virus is required, we omitted the overnight pre-incubation of virus and PEGylated nanorod for this measurement.

### Potential Mechanisms of Plasmonic Enhancement

What is the mechanism underlying the plasmonic enhancement of virus inactivation? The fact that the enhancement does not require a physical contact between the nanorod and the virus particle argues against field-enhanced ISRS or multiphoton absorption effects as underlying mechanisms. Both of these effects are strongly E-field dependent and the E-field intensity decays rapidly with separation from the nanorod surface. At a distance of 100 nm, the E-field has already decayed to the value of the incident light field. Considering the nanorod concentration used in this work of ~1 × 10^11^ particles/mL, which corresponds to one rod in a cube of solvent with side length of approximately 2 μm, we can exclude that virus particles and nanorods co-localize close enough to generate a sufficient E-field enhancement to impact ISRS or multiphoton absorption of the virus. For similar reasons, it is unlikely that thermal effects are the underlying cause of the observed virus inactivation. Although the resonant fs laser excitation induces a temperature jump in the nanorod and its immediate environment, this effect is local (Figure S2) and a subsequent thermalization with the heat bath of the solvent rapidly abrogates any temperature gradients. The measured temperature fluctuations during the course of our experiments were <3 °C.

**Figure 5:**
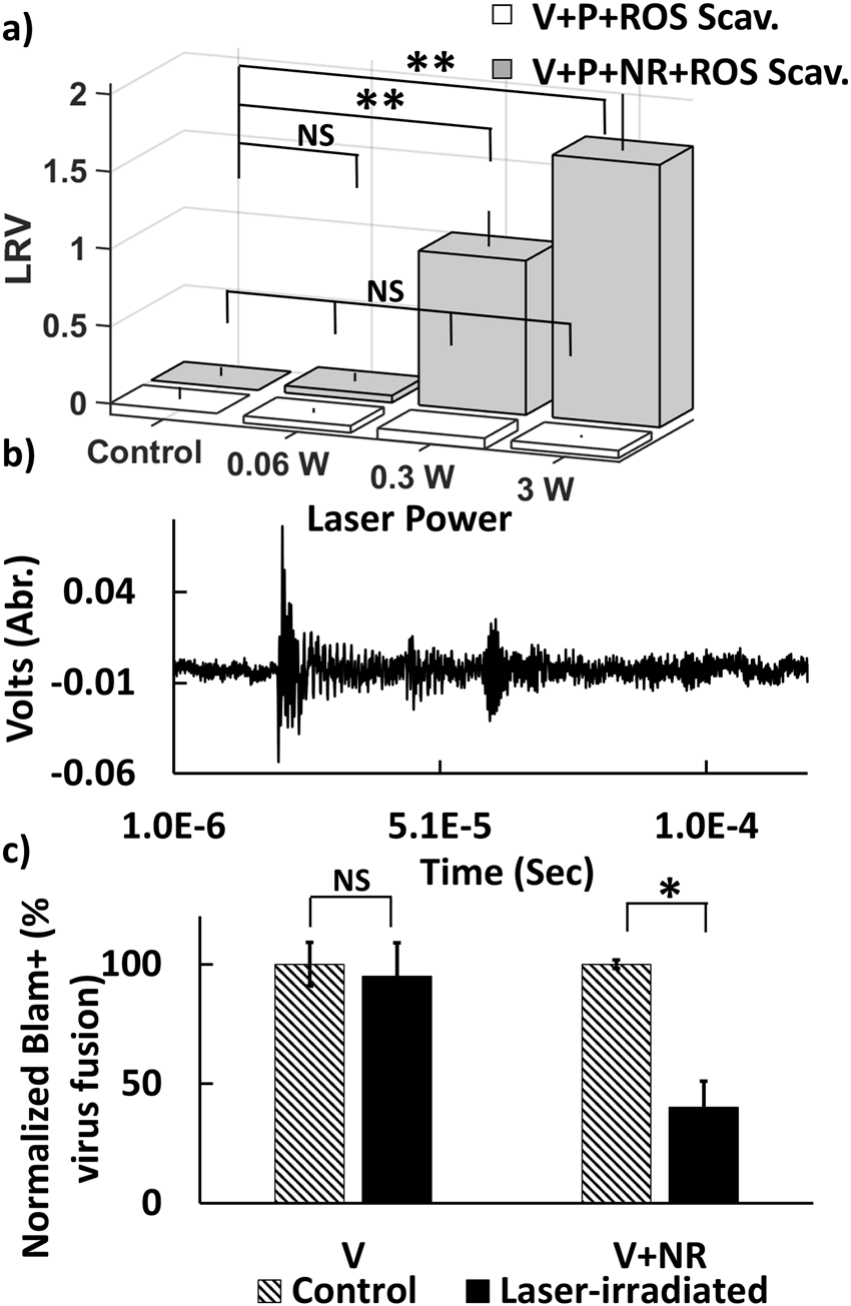
(**a**) LRV obtained after 10 s irradiation with pulsed 805 nm laser as function of average power for virus + ROS scavenger mix (white) and virus + PEGylated nanorod + scavenger mix (gray). All samples contained IgG hybridoma (P). The scavenger does not suppress viral inactivation. (**b**) Ultrasonic wave emitted from 0.3 W laser irradiated nanorods recorded by photoacoustic transducer. (**c**) Relative virus fusion (normalized by no treatment control) obtained after 10 s irradiation with pulsed 805 nm laser with 3 W. Laser exposure in the presence of nanorods reduces viral fusion.

One alternative mode of virus inactivation that does not require direct contact between nanorods and virus is the light induced generation of reactive oxygen species (ROS). It has been demonstrated in previous studies that plasmon excitation in gold nanocubes can generate singlet oxygen (^1^O_2_), superoxide anion (O_2_^−^), or hydroxyl radical (^•^OH)^39^. Even if their formation is limited to the immediate vicinity of the nanorods, the ROS can diffuse in solution. Singlet oxygen, for instance, has a typical diffusion length in water of approximately 200 nm^40^. If these reactive species result in the disabling of important surface groups required for binding to and uptake into host cells, they could – in principle – account for the observed MLV inactivation. To test whether these ROS were involved in the virus inactivation, we repeated the fs laser irradiation of a mix of MLV and resonant nanorods in the presence of ROS scavengers. In particular, we used sodium azide (scavenger for ^1^O_2_), MnTBAP (scavenger for O_2_^−^), mannitol (scavenger for ^•^OH)^41,42^. While the concentration of ROS scavengers used in these experiments did not show any cytotoxicity (data not shown), addition of ROS scavengers did not suppress nanorod mediated virus inactivation (Fig. 5a), suggesting that ROS formation mechanism is not responsible for the virus inactivation.

Another potential virus inactivation mechanism that does not require direct contact between the virus and the nanorods is plasmonically-enhanced shockwave generation. The duration of the incident pulse is crucial in determining the feasibility of inactivation^43,44^. Intense pressure wave generation is expected to dominate only for very short pulse durations as used in this work. We emphasize that although Tsen and coworkers dismissed the pressure wave argument in their studies due to insufficient laser power, the situation is fundamentally different in our case due to the presence of the plasmonic nanoparticles, which we expect to greatly reduce the power threshold for shockwave generation. Based on published analyses of threshold intensities in the presence of nanorods^45^, we estimate that average powers ≥0.3 W are sufficient to trigger shockwaves under our experimental conditions using pulse durations of 35 fs. We recorded the photoacoustic response of the nanorods, which represent a broad band acoustic emissions followed by a gradual fluctuation that decreased to near zero until another distinct oscillation arrives (Fig. 5b). Although the temporal resolution of our experiments (our transducer was limited to a frequency of 2.5 MHz) is insufficient to directly resolve shock waves, the successful detection of the acoustic emissions does confirm an efficient transfer of optical energy provided by the incident laser to the resonant nanorods. As the shockwave mechanism is consistent with all experimental observations, we propose it as underlying mechanism of the observed virus inactivation. Future studies with higher temporal resolution will be able to shed more light on the shock wave generation and its relevance for MLV inactivation.

Independent of the exact physical mechanism that causes the plasmonic enhancement of virus inactivation, it is key to establish which stage of the viral infection is affected by it. Since TEM inspections after laser exposure did not reveal obvious morphological changes in the virus samples, we hypothesized that the effect is primarily caused by molecular-level perturbations of the MLV membrane and/or surface groups, which prevents an effective binding to the host cell and subsequent fusion. To test this hypothesis, we repeated the fs laser irradiation of MLV/luc particles containing S15-BlaM fusion protein in presence and absence of resonant nanorod. Figure 5c shows the normalized virus fusion percentage for the virus and a virus-nanorod mixture, without (control) and with laser irradiation at an average laser power of 3 W for 10 sec. The experiments show that only laser irradiation in the presence of resonant nanorods achieves a measurable decrease (~2.5-fold) in virus fusion. Laser irradiation without nanorods had no measurable effect on fusion. These data corroborate the hypothesis that the plasmonically enhanced photonic inactivation primarily inactivates viral functions responsible for early stages of the infection, including host cell binding and fusion.

## Conclusions

We have demonstrated the enhancement of fs-pulsed NIR radiation induced photonic virus inactivation through plasmonic nanoparticles. Irradiation in the presence of resonant nanorods significantly reduced viral fusion with the host cell, suggesting that the plasmonic enhancement acts on viral surface functionalities responsible for early stages of the viral infection rather than on the viral genome or enzymes. LRVs of ≥3.7 were achieved with MLV in as little as 10 s of irradiation. The inactivation was highly specific to the virus while co-incubated IgG antibodies did not show any loss in functionality. The ability to enhance the efficacy of selective photonic virus inactivation with NIR radiation through plasmonic nanoparticles paves the path to a broader applicability of the technique in research and technology.

## Electronic supplementary material

Supplementary Information
