# Supplementary material for: Plasmonic Enhancement of Selective Photonic Virus Inactivation
Source: Sci Rep. 2017 Sep 20;7:11951. doi: 10.1038/s41598-017-12377-5 (PMC5607298; doi:10.1038/s41598-017-12377-5)
Supplement: Supplementary file 1 — Supplementary Information [file 41598_2017_12377_MOESM1_ESM.pdf]

Plasmonic Enhancement of Selective Photonic Virus Inactivation

Mina Nazari,[#,¶] *Min Xi,*[#,‡] *Sarah Lerch,*[#,‡] *M.H. Alizadeh,*[#,‡] *Chelsea Ettinger,*[§] *Hisashi Akiyama,*[§] *Christopher Gillespie,*[&] *Suryaram Gummuluru,*[§] *Shyamsunder Erramilli,*[#,†,*] *Björn M. Reinhard*[#,‡,*]

Departments of [¶]Electrical and Computer Engineering, [†]Physics, [‡]Chemistry, and [#]The Photonics Center, Boston University, Boston, MA 02446, United States

[§]Department of Microbiology, Boston University School of Medicine, Boston, MA 02118, United States

[&] Next Generation Bioprocessing , Millipore-Sigma, Bedford, MA 01730, United States

*E-mail: shyam@bu.edu and bmr@bu.edu

SUPPORTING INFORMATION

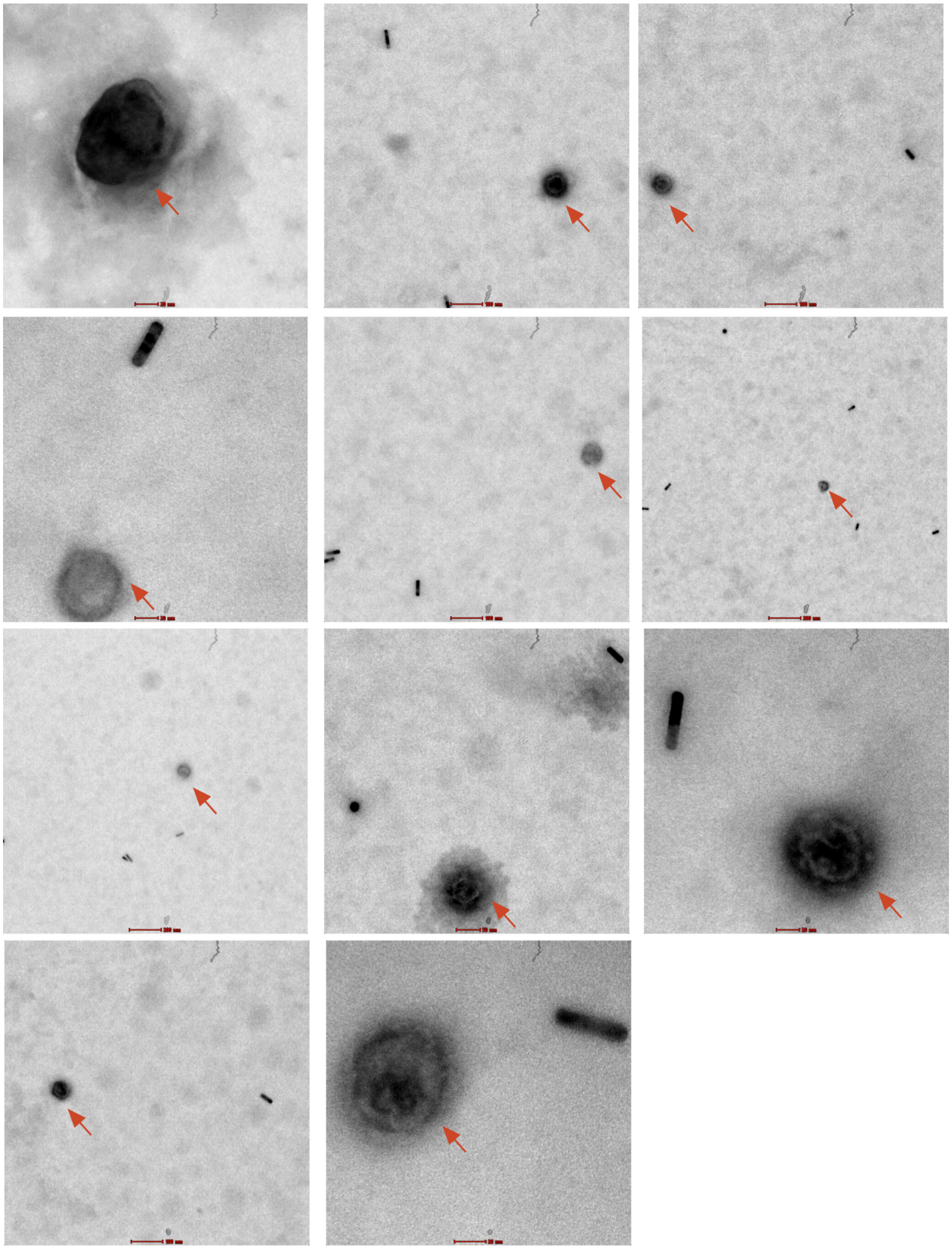

Figure S1: TEM image of MLV particles after exposure to 35 fs laser pulses for 10 s. Laser power was 3 W. Images were acquired at different magnifications after staining with OsO_4. Virus particles are marked with red arrows.

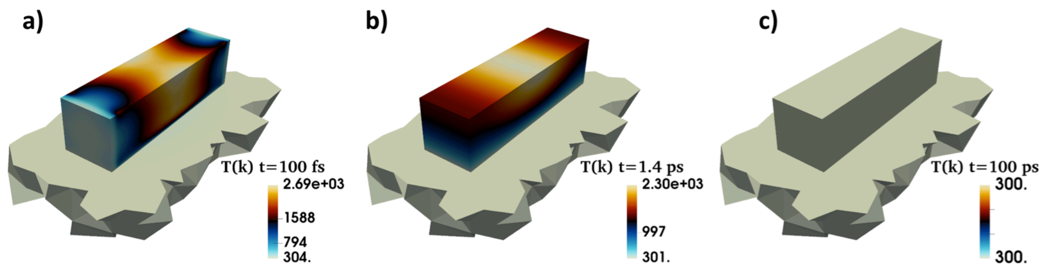

Figure S2: According to the heat map simulations of the gold nanorod excited by fs laser at t=50 fs. There is a time dependent temperature gradients inside the nanorod (a) t=100 fs, (b) t=1.4 ps, (c) t=100 ps. We used heat equation to find the temperature distribution for the nanorod on a glass substrate. In the first step, the source function, which is the absorbed optical power as a function of spatial coordinates is calculated using EM simulations. Then it is used as the source for the heat equation. For the boundary conditions we assume boundaries of fixed temperature (300 k) far from the nanorods.

ADDITIONAL METHODS:

TEM Characterization:

Electron microscopy samples were prepared on carbon-coated TEM grids. For characterizing gold nanorods, 5 μL of the nanorod sample, both before and after laser irradiation, were dropped onto the TEM grids. After all liquid evaporated at room temperature the grids were stored in vacuum before imaging using TEM microscope.

Mixtures of virus particles and PEGylated nanorods were also characterized after 10 s laser exposure with 3 W. This sample which contains viruses, went through a fixation and negative staining process. Because of the large excess of PEGylated nanorods compared to the viruses, we spun down the PEGylated nanorods by centrifuging the solution at 14k rpm for 10 min. A volume of 5 ul of the supernatant of the sample was initially incubated on TEM grid for 30 min. Then excess solution was removed by a clean filter paper. Then the samples were incubated with 2.5 % glutaraldehyde (Sigma-Aldrich) in 1x PBS for 10 min and subsequently the grid was washed with 1x PBS three times. Then the sample was incubated with 1% OsO_4 in PBS for 10 min. The sample was washed with water three times and then negatively stained with 1 % $Na_3P(W_3O_{10})_4$ in water for 10 sec. Finally, excess solution was removed by blotting the sample and the grid was stored in vacuum and imaged using TEM microscope (Tecnai Osiris, FEI).
